# A preliminary research on transcultural capacity in global public health: from the view of public health professionals

**DOI:** 10.1186/s12889-023-15312-8

**Published:** 2023-03-10

**Authors:** Feng Ning, Liu Xin, Wang Quan, Liang Xiaohui, Dong Xiaoping

**Affiliations:** 1 Center for Global Public Health, Chinese Center for Disease Control and Prevention, Room 211, 155 Changbai Road, Changping District, 102206 Beijing, P. R. China; 2 Jiangsu Provincial Center for Disease Control and Prevention, 122 Maihua Road, Qixia District, 210028 Nanjing, Jiangsu Province China; 3 School of Public Health, Wuhan University, 115 Donghu Road, Wuchang District, 430078 Wuhan, Hubei Province P. R. China; 4 National Institute for Viral Disease Control and Prevention, Chinese Center for Disease Control and Prevention, 155 Changbai Road, Changping District, 102206 Beijing, P. R. China

**Keywords:** Transcultural capacity, Global public health, Assistance cooperation, Public health professionals, Cultural diversity

## Abstract

**Background:**

Transcultural capacity is a key component of consolidated global public health assistance cooperation (GPHAC). The aim of this study is to investigate the transcultural capacity perceptions of public health professionals from China’s disease control and prevention system after relative training in order to provide a reference for enhancing transcultural capacity during the practice of GPHAC.

**Methods:**

A cross sectional qualitative survey in which self-administrated questionnaire with 5 open ended questions was used. The questionnaire was disseminated on the completion of an online training for China’s senior public health professions on transcultural capacity in GPHAC. Descriptive statistics, word frequency analysis and content analysis were used to analyze the questionnaire data.

**Results:**

Totally, 45 participants took part in this training, 25 of them voluntarily participated in this survey. The participants demonstrated the need for transcultural competence in public health services and suggested improvement in the course content arising from their wealth of knowledge and practical experience in the field. 96% of the participants considered that the training course was “very necessary” and “meaningful”. The most interested topics were “Overview of transcultural adaptation and GPHAC”, “Transcultural adaptation and response” and “African culture and health”. The contents about “Country-specific analysis on cultural factors in public health”, “rapid transcultural adaptation” and “more specific practical experiences in diverse cultural backgrounds” were suggested to be added in future training. The participants considered that transcultural capacity ensured the smooth progress of GPHAC and they both could complement each other, transcultural adaptation was the premise of gaining trust and reaching cooperation, it can be conducive to the health assistance professionals to integrate into local cultural life, facilitating their foreign assistance work to be effective and efficient, and impart experiences well. The participants hoped to put the concept into action.

**Conclusion:**

The importance of transcultural competence in GPHAC is becoming a consensus of public health professionals. Enhanced transcultural competence reflected in the attitude of public health as well as other health workers would promote GPHAC and would foster efficient emergency health response management among many countries.

## Background

Transcultural capacity as a component of global health practices has gained focus in recent times due to trend of globalization and social network which widens people’s horizon on global health practices. Training on transcultural capacity has been widely applied to health professionals, healthcare and healthcare policies [1, 2].

The transcultural capacity building in China was initiated, targeted and incorporated into the global public health capacity building program from the year 2018 [3]. This project spanned through the establishment of guidelines, collection and exchange of transcultural experiences in global public health practices, curriculum development, and organizing of trainings which is targeted towards quintessence of global public health as cross-border, multi-sector and multidiscipline actions in health [4]. Initially, the establishing of guidelines for transcultural capacity and its incorporation as part of capacity building in public health, stems from the experience of the authors who participated in the Ebola virus disease (EVD) response in Western Africa. The authors provided useful information on disease control from anthropologists’ recommendations spanning from safe burial to safe and dignified burial measures for deceased EVD patients, but critically reliant on cultural consideration perspective [5].

The transcultural issues reflected in the action of fighting against Ebola promoted the initiation of the transcultural capacity building activity which was expected to equip health professionals working in China’s public health system, with adequate knowledge about transcultural capacity that would facilitate their emergency response to health issues in foreign countries. This would invariably help them to achieve teamwork with their foreign colleagues during technically supportive work in foreign countries and as well when foreign health professionals are in China on health missions.

These efforts to establish transcultural capacity building activity had gained interest and active participations of both multidisciplinary experts and public health professionals. A guideline on transcultural capacity in global public health cooperation was developed and the authors defined “transcultural adaptation” as “changes in the physical process and mental responses which an individual undergoes when he/she embraces a new culture due to contact with other people”.

Since 1963, China has dispatched foreign aid medical team members to developing countries in an extensive manner [3], and the transcultural adaptation of medical team for foreign aid has been studied [7]. Transcultural researches in health fields are comparatively more common in transcultural competence and self-efficacy as well as training model in nurses and mental health service providers who offer health services to their clients/patients who are of multicultural backgrounds or identity. In same lieu, there are relatively more studies on the transcultural adaptation of international students (Chinese students going abroad or foreign students going to China), personnel of multinational corporations and foreign trade, but relatively fewer studies on personnel of public health foreign aid [8].

However, researches on disease control and epidemiology survey from trans-culture perspective are still limited. In recent times, trans-culture was introduced into epidemiological survey for the first time. Four dimensions of transcultural competence (awareness and reflexivity, cultural and structural validation, sensitivity and representativeness) were considered in three stages [12]. In another study, the author suggested that cultural training in health professionals produced very little effect [9]. In China’s international cooperative program, transcultural awareness has been strengthened in the maternal and child health promotion and service delivery when targeting various ethnic minority service objects [10]. With the increase of dispatch of public health personnel to foreign countries by China, special transcultural study on global public health assistance and cooperation (GPHAC) is becoming more and more meaningful.

In December of 2021, a two-day’s online transcultural training course was delivered accordingly by multidisciplinary teachers in global health, disease control and prevention, culture, medical ethics, sociology etc. from both universities and centers for disease control and prevention (CDCs) in China. The trainee participants were senior public health professionals working in China’s national and provincial CDCs who had been engaged in or were intentionally committed to foreign assistance and cooperation work in public health. Besides personnel capacity development, the training targeted to strengthen the interaction networks among the program developers, teachers and trainees, incorporate new and practical experiences of the trainees into teaching materials to meet up with the learning needs and as well review it from time to time. On the completion of the training course, a qualitative survey by using open ended questions in questionnaire was conducted in the participants to understand their perceptions on the role of transcultural capacity in global public health. The investigation results were reported in this article. The aim of this study is to investigate the transcultural capacity perceptions of public health professionals from China’s disease control and prevention system under the premise of receiving the online training in order to provide a reference for enhancing transcultural capacity in GPHAC practice.

## Methods

### Study design

This is a cross sectional qualitative survey in which self-administrated questionnaire with selective questions and open ended questions was used. The questionnaire was disseminated and collected back through an online medium. There were five open-ended questions in the questionnaire: (i) What is your opinion on role and necessity of the training course to promote your current work? (ii) What are your most interested topics of the training course? (iii) What content do you think that are necessary to be added into the training curriculum? (iv) What are your main attainments from the training course? (v) How about your opinions on the role of transcultural capacity in global public health cooperation?

The aim and content of the survey were explained to the participants before the survey. The participants’ verbal consent was obtained. Meanwhile, at the beginning of the questionnaire, there was a special text to inform the participants: “The researchers will keep the participants’ answers to the questionnaire confidential, not disclose personal information of the participants, and not disclose the answers corresponding to the personal information of the participants. The information and answers collected will be used only for group analysis. Participants are considered as informed consent if they answered questions. Verbal consent was obtained from the participants for this questionnaire survey. Participation in the questionnaire is voluntary, can be suspended at any time. The participants who didn’t agree with the questionnaire would quit on their own.”

### The main contents of the training course

**Fig. 1:**
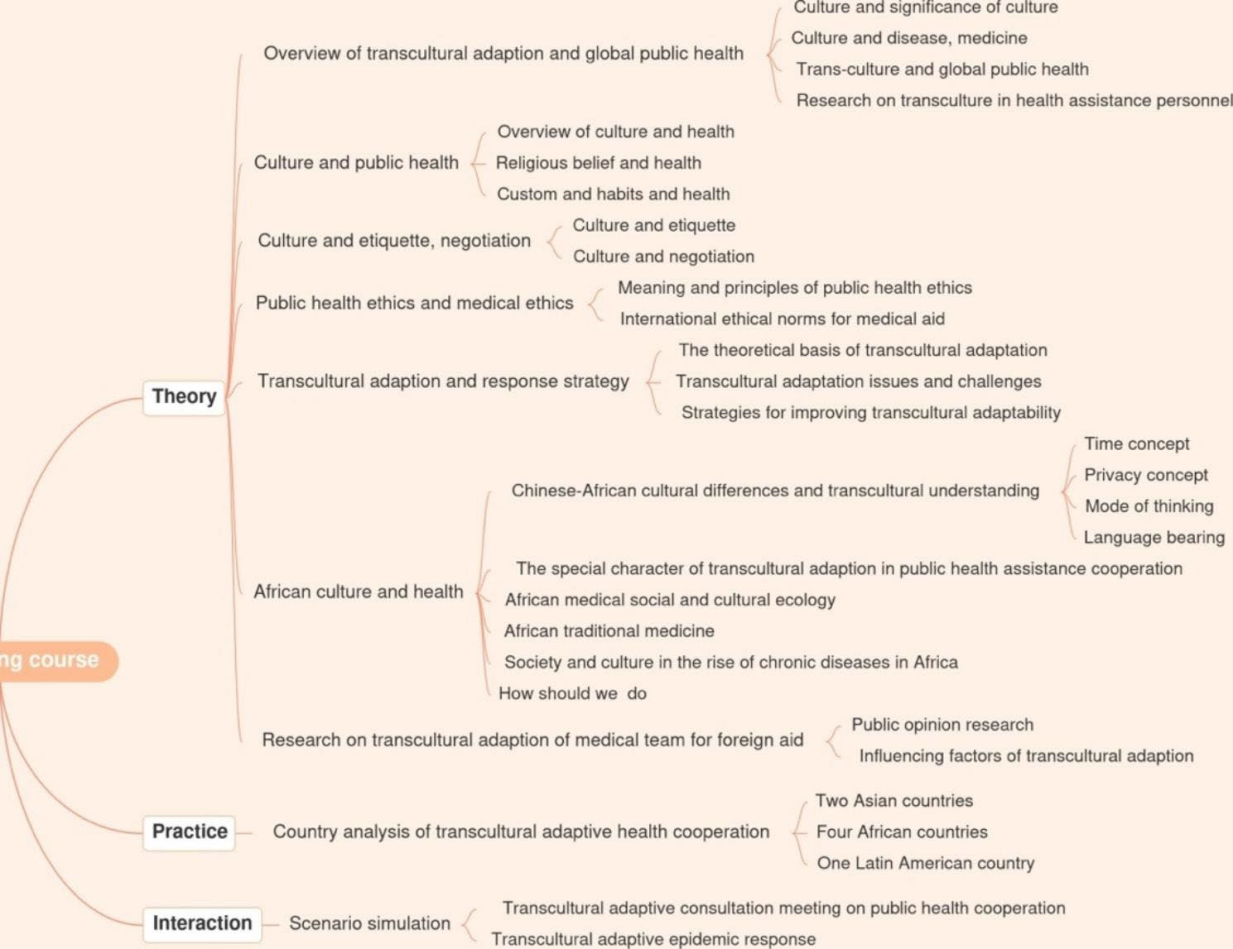
The mind map of the training course

The training course consisted of three parts: theory, practice and interaction (see Fig. 1).

(i) The theory part was comprised of seven topics: Overview of transcultural adaptation and global public health, Transcultural adaption and response strategy, Culture and public health, Culture and etiquette as well as negotiation, Medical and Public health ethics, African culture and health, and Transcultural adaptation research for medical team members on foreign aid. (ii) The practice part entailed: Country analysis of transcultural adaptive health cooperation that was delivered by health experts who had working experiences about providing technical support for epidemic response and health system surveys in seven countries from Asia, Africa and Latin America. (iii) The interaction part was scenario simulation. Prior to the online training which was used for this study, the first and second parts were initialized where discussion and consultation meetings were conducted among teachers for the course preparation.

### Study subjects

The training notice was issued to China’s national to provincial CDCs through office automatic (OA) system to invite senior public health professionals who had been engaged in or intentionally committed to foreign assistance and cooperation work in public health to participate.

### Data collection and analysis

On the completion of the training course, the questionnaire structured by the researcher was sent to the participants through WeChat group which is a kind of social media just like Facebook. The participants on individual basis filled the questionnaires anonymously and returned it to the researcher within 3 days after the training course through emails or WeChat group. The anonymous filled questionnaire was used for descriptive statistics and content analysis. The information of selective questions was mainly analyzed by descriptive statistics and content analysis, Content analysis was used to analyze the participants’ main opinions about transcultural capacity in global public health.

### Word frequency analysis

Word frequency analysis is a very popular text analysis method in recent years. This method calculates the frequency of each character or word in the text by disassembling the input text. It is generally believed that the higher the frequency of word occurrence, the more important the content is in the text. The frequency of notional word in a particular text usually can reflect the text hotspots and main opinions. With regard to the main opinion of the participants, word frequency analysis was conducted using the software from the website of NiucoData (http://cloud.niucodata.com/). The top six highest frequency words were shown in the result. The answer for open questions was mainly analyzed by word frequency analysis.

## Results

Totally, 45 participants signed up the training course, among them, 25 participants voluntarily participated in the survey. The questions were about the participants’ opinions on role and necessity of the training course to promote their current work, the utmost priority area for the participants in the training courses, the contents necessary to be added, the participants’ main attainments from the training course and their opinions on the role of transcultural capacity in GPHAC.

### The necessities and significance of the training

96% of the participants considered that the training course was “very necessary” (24/25), and one thought it “necessary”. Most of the participants (24/25) considered that the training course was “very meaningful” (more than half) or “meaningful” to promote their current work. All of the participants were “very satisfied” with the organization of the training.

*“It is very meaningful and necessary to hold such a training course. I think it is well organized. The experts invited are both special in foreign exchanges and culture, and have special experiences in on-site epidemic response. The content and information are very rich, vivid and attractive that is instructive for our future work.” “It is necessary to strengthen the transcultural capacity building and psychological counseling, which can help to adapt to the local culture more quickly after arriving abroad, better integrate into the local life, and improve psychological adaptability.”*

### The most interested topics of the training

For more than half of the participants, the most interested topics were “Overview of transcultural adaptation and global public health” (52%, 13/25), “Transcultural adaptation and response” (64%, 16/25) and “African culture and health” (56%, 14/25). Nearly half of the participants were the most interested in the topics such as “Culture and etiquette, negotiation” (44%, 11/25) and “Country analysis on transcultural adaptive health cooperation” (44%, 11/25).

*“The lectures of all the experts were very wonderful which were of great guiding significances for our future work. I especially like the country analysis part of transcultural adaptation to health cooperation, which has condensed the experiences of public health cooperation in different countries and benefits us a lot.”*

### Recommended additions of the training

More than half of the participants believed that “Transcultural adaptation and response” (52%, 13/25) needed more detailed introduction. Besides, the participants recommended additions in these aspects.

Firstly, the participants mentioned to add the content about country-specific analysis on cultural factors in public health.

*“It is suggested to make the analysis on more countries about their cultural characters related to public health because of China’s closer cooperation with the world in public health, to invite more teachers including those from international organizations and developed countries with the fruitful experiences about GPHAC to give lectures on the topics such as the cultural issues in the control and prevention of tropical diseases, the public health problems pertaining to cultural backgrounds of countries, the difference and application of different cultures and etiquette .”“It is needed to consider the multilateral transcultural issues during public health cooperation. It is beneficial to analyze and refer to the transcultural adaption of other countries in their GPHAC.”*

Secondly, the participants hoped to add the content on how to rapidly overcome cultural shock.

*“The vast majority of us are working in disease control, dealing with the epidemic of diseases. So, we would like to learn how to integrate with the local medical teams and mobilize various resources quickly to respond to the epidemic.”*

Additionally, the participants were very interested in deeper participation in the interactive training on transcultural competence and hoped to have face to face lectures, increase the case explanation, cases of global public health emergency response, and more specific practical experiences on medical tasks and international public health projects.

*“I hope to learn more case studies, especially for the cases of transcultural health aid including the type of aid, structure of the aid team, the implementation process, the challenges that occurred and final evaluation etc.” “It would be better to increase the proportion of transcultural practice and application so that the participants can increase their understanding through discussion and exercise.”*

### The main attainments of the training

Participants generally believed that they got a rich harvest in knowledge, concept, vision broadening, and importance etc.

**Fig. 2:**
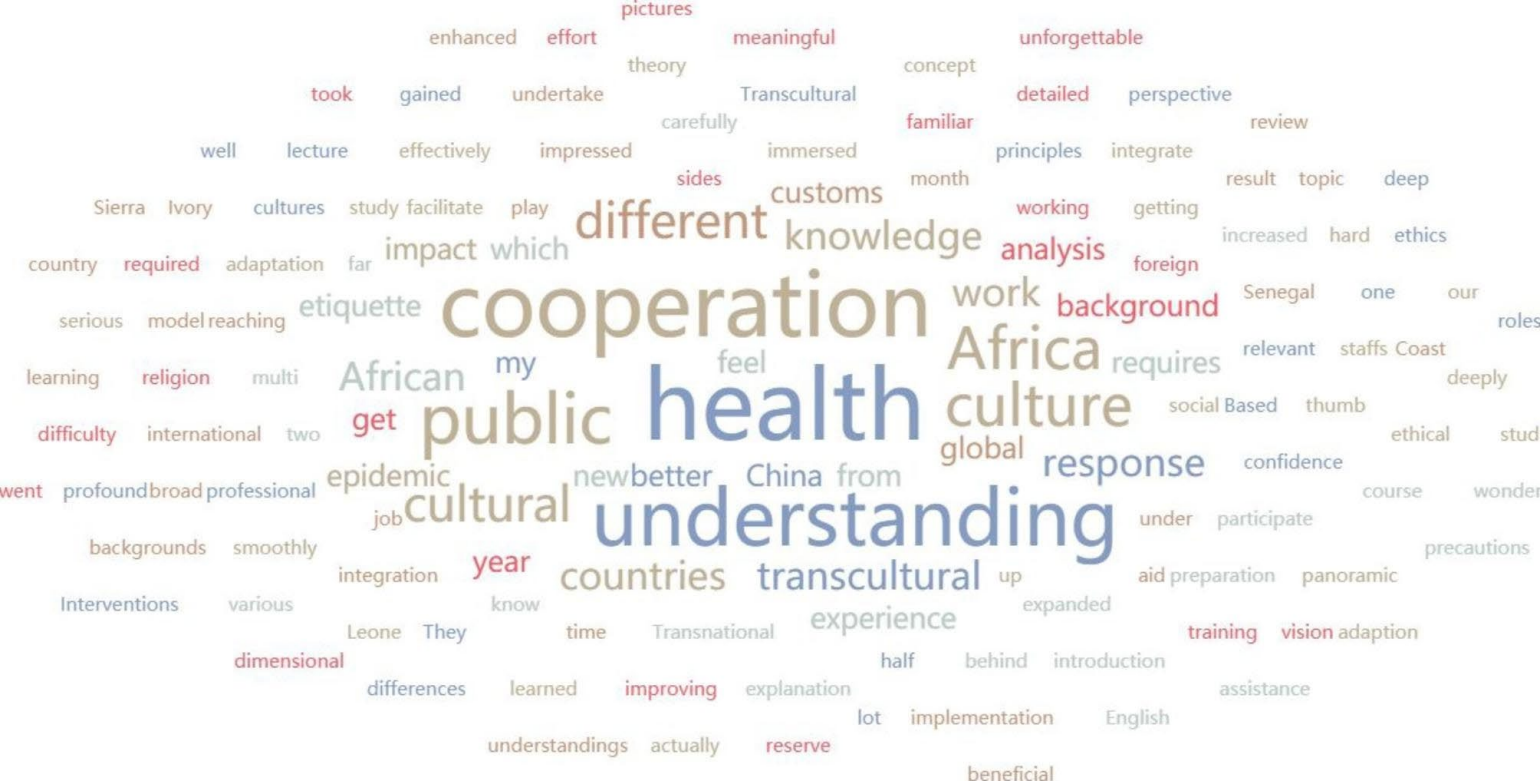
Notional word frequency cloud of the participants’ attainment statements

*“The training is systematic, vivid and interesting. We have gained a lot”*. The most frequent used notional words in the participants’ statements were “health”, “cooperation”, “Africa”, “public”, “understanding” and “culture” etc. (see Fig. 2).

Firstly, the participants thought their knowledge and interest in the transcultural topics of GPHAC had increased.

*“I have gained new understandings of transcultural knowledge including the concept, theory, model and response in GPHAC, increased the reserve of relevant knowledge, and expanded the vision.” “I know more about the precautions of foreign culture, etiquette and international work, have a detailed understanding of the social and cultural background, and ethical principles of different countries, cooperation experiences and code of ethics”. “I become more interested in this topic, getting to know that the foreign health aid personnel are very hard and face multiple difficulties.” “I have got a comprehensive understanding on the significance of transcultural exchanging among governments, Africa culture and health, and transcultural adaption issues plaguing foreign aid health team etc.”*

Secondly, the participants have recognized the importance of transcultural capacity in GPHAC. In particularly, it is of great significance for foreign aid personnel to better carry out their work.

*“Transcultural adaptation is a serious topic. Only by improving our quality and understanding on the impact of cultural differences on public health can we do a better job. Transnational cooperation requires to not only learning professional knowledge and foreign language such as English well, but also getting familiar with various customs and cultures of different countries, so that the cooperation can get twice the result with half the effort. The impact of culture on health is far reaching, while it is required a deep understanding of the cultural background behind it for effective interventions, and we can actually do more.”*

Thirdly, combining with experiences of one’s own, the participants generated resonance to increase their enthusiasm.

*“I went to two African countries last year to participate in a one-month epidemic response. I feel deeply about the introduction on transcultural analysis on the countries with a lot of pictures which immersed me and took me back to the unforgettable past time after a year. Transcultural adaption is very meaningful.” “The country analysis was wonderful so as to make me get a multi-dimensional understanding of various countries. The panoramic explanation and review impressed me very much as I have been working in Africa. Combined with their years of research, the teachers have systematically explained the basic characteristics of public health assistance and cooperation projects, the social and cultural gap that needs to be faced, and the multi-faceted characteristics of medical or public health, such as dynamics and complexity, so as to give us a steelyard for how to work abroad.”*

### Perceptions on transcultural capacity

**Fig. 3:**
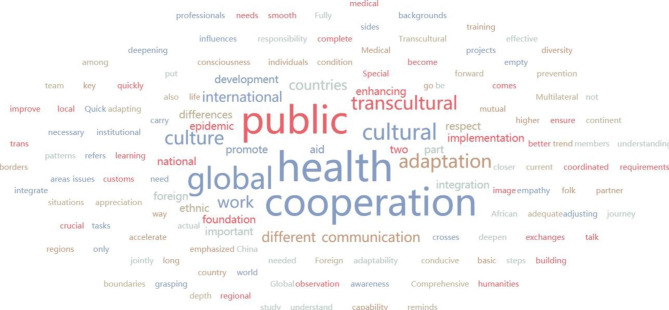
Notional word frequency cloud of the participants’ perception statements

The first result of perception on transcultural capacity is about the necessity and significance of transcultural capacity. The most frequent used notional words in the participants’ statements were “health”, “cooperation”, “Africa”, “public”, “understanding” and “transcultural” etc. (see Fig. 3).

The participants considered that transcultural capacity ensured the smooth progress of GPHAC and they both could complement each other. Especially, some of the participants illustrated this from the definitions of trans-culture and global health. The participants believed that transcultural adaptation is the premise of gaining trust and reaching cooperation. It can better promote global public health cooperation, be conducive for the health assistance professionals to integrate into local cultural life, facilitating their foreign assistance work to be effective and efficient.

*“A smooth GPHAC needs a good understanding of cultural and institutional differences, adequate transcultural adaptation and capability building for public health professionals from different cultural backgrounds.” “Most of the time, we just continued the conventional practices and lacked an in-depth understanding and analysis of the culture of the partner countries. Foreign aid is a journey of epidemic prevention and cultural integration. From observation, respect and appreciation to integration and empathy, are the bases for a good public health cooperation”. Quick adapting to culture and adjusting communication as well as work patterns are key steps.”*

*“The basis of GPHAC lies in communications and exchanges between the two sides. Fully appreciating the national situations, culture and folk customs of the partner are crucial and will be conducive to deepen the coordinated implementation of cooperation projects on disease control and prevention.”*

The second one is about the significance of transcultural capacity building. The participants considered that learning transcultural adaptation is helpful to carry out GPHAC and impart experiences effectively. They hoped to put the concept into action.

*“Comprehensive and in-depth study on trans-cultural is meaningful to improve awareness and adaptability. The current global epidemic reminds us of the need for closer international cooperation. Special cross regional and transcultural training is very necessary to accelerate transcultural adaptation and promote global public health cooperation. Transcultural adaptation in GPHAC cannot be empty talk. In the actual work, the consciousness and effective implementation are responsibility.”*

## Discussion

The main focus of the study is to investigate the perceptions of senior public health professionals from China’s disease control and prevention system on transcultural capacity, aiming to strengthen transcultural capacity that is one of the best health practices and a key component of global health program for more consolidated GPHAC. This is the first study to conduct a systematic training and research within China’s public health system on transcultural capacity for GPHAC. The views of the subjects were based on the training which was not only focused on the sensitization of participants, but also geared towards the improvement of training and teaching resources via constructive interaction between the teacher and participants.

The findings demonstrated that the interest and attention on transcultural capacity have gained the focus of public health professionals. They perceived that the relationship between transcultural capacity and GPHAC are intertwined and move in tandem with each other. The participants proposed to take the initiative to strengthen and display transcultural capability in global public health practices. They also displayed their views on macro perceptive of transcultural capacity with regards to global public health, which involves the health assistance work, win-win cooperation, and the local customs of the country etc. All these are encompassed in the tenets of public health global practices but did not limit in the individuals’ skills for health services.

The results of the study showed that the participants were satisfied with the content of training courses which adequately covered the main topics about the transcultural capacity in GPHAC. The participants proposed points of interest and suggested that teaching hours for such trainings be extended, in order to meet up with the practical needs of transcultural building capacity. Meanwhile, they submitted that in the practice parts, permissions should be made for understanding interdisciplinary knowledge including social development and economic knowledge from cultural perspective and its critical role.

Although the participants were exposed to training on transcultural capacity, they believed that the short-term training may not be sufficient to understand and actualize the objective of transcultural building in global health practices. They pinpointed the beneficial impact of transcultural adaptation on GPHAC, an indication that transcultural adaptation is a continuous process that requires cooperation of all partners involved at all times. This communication channel is of relevance to all parties irrespective of individuals, groups or institutions and of critical value to participants who have engaged in GPHAC.

While, the participants showed less interest in the theory part of the training, they believed that the theoretical explanations appeared more abstract than practical that illustrated that the adoption of actual case studies to elucidate theoretical basis in future trainings would be more useful to the participants. It is then speculated that the theory part of the training will be more relevant when experts are rich in experience and knowledge about transcultural capacity from perspective of global public health practices.

The theory of social relations of the audience postulates that the selective response behavior and interpretation of the information transmitted by the audience are affected by their social relations [11]. In this context, the participants are regarded as audiences and the training as communication activity while the social relations are considered as their involvement in China’s public health system as senior health professionals who had been or committed to public health assistance and cooperation. Thus, the course contents approved by the participants and their proposed additional contents reflect the needs of their social relations and can be used as the basis for further curriculum improvement.

Previous researches [2, 9, 12, 13] demonstrated the crucial need for public health professionals to show skills that reflected flexibility and unprejudiced to intercultural competence. Some other research proposed five principles: transcultural education plan and transcultural competence, a framework including adaptation and self-evaluation on cultural diversity, and the cultural component variation [14]. In the present research, the participants showed a strong enthusiastic attitude towards understanding the cultural diversity of various countries from a multi-dimensional perspective and hoped to acquire more experience to be shared on the professional networks. Therefore, from the afore mentioned findings and theories, we speculate that the key element of transcultural capacity building for public health professionals, is to have an expertise rich knowledge of cultural diversity and carry out practice-based reflection of such.

Moreover, transcultural capacity in global public health is not limited to individual oriented skills but encompasses development measures for group, multi-sectoral and international work from a macro perspective. It also takes into consideration the impact of larger variables such as environment, facilities, standardized working language and public health services/interventions/actions and so on from a holistic view as components in transcultural adaptation process.

A few of transcultural studies in the field of health suggested that transcultural competence was critical to improve the quality of healthcare services and its efficacy could be considered as a strategy to ensure optimal healthcare services at an effectual capacity [6, 12, 15–20]. Previous researches [21, 22) have affirmed the relevance and the significance of transcultural competence in disease prevention and control. All these were suggestive that trainings on transcultural competence were generally recognized and acceptable by healthcare professionals.

Furthermore, attainment of transcultural competence among health professional globally may require that, components of transcultural capacity building and competence are reflected in the medical and public health education curriculum. A previous study [1] demonstrated the conspicuous positive synergistic effect observed when transcultural competence was incorporated into medical education and global public health practice rather than a single entity. The health practitioners observed that cultural awareness-based approaches were more effective in handling transcultural competence and lead to offer of better health services to the public with existing cultural diversity. Such approach provided room for an active engaging-community which facilitated integration of multiple institutional levels, multi-disciplinary expert team including health, social sciences with cultural diversity to establish a consensus [1, 14, 16, 23]. Similarly, our training in this research was multidisciplinary approach-based. The participants reached a consensus on the necessity of transcultural capacity as a pivotal tool to successful global public health cooperation. In particular, such capacity is relevant to global public health practices spanning from individual level, professional level and self-development.

Several previous researches [2, 24, 25] have explored the dimensions and scales to evaluate the transcultural competence in healthcare professionals. In addition, some authors [26, 27] proposed that transcultural approaches could be enhanced via the utilization of educational tools and resources for favorable learning environment, adoption of active learning strategies, experiential learning opportunities, teacher modeling, and internet-based learning. Commonly, research on trans-culture in the field of health mainly focus on the cultural competence in individual medical services and others several investigated the situations in epidemiology or population services. Whereas, systematic explorations on the transcultural capacity in global public health cooperation from the perspective of multi-sectoral, international and multi-disciplinary public health cooperation are rare, especially for the improvement of transcultural capacity in group oriented public health services globally. Thus, it becomes expedient to carry out active interaction and discussion with global health partners and public health professionals continuously, in order to factor rising global trends into consideration of key elements of transcultural capacity in global public health.

This study has been able to narrow this research gap. Our findings in relation to previous ones would provide a stronger baseline for a standardized transcultural capacity building in GPHAC. This would enhance health services and practices that reflects transcultural competence among health professionals, which finally promotes effective GPHAC. There are limitations to this study, the number of respondents involved in the cross sectional survey is relatively small while with the in-depth work conducted in related areas, information from more subjects will be collected continuously to refine the findings.

## Conclusion

Meeting up with increasing demands for global public health practices, transcultural appropriate has gained much attention among China’s public health professionals who have received the training on transcultural capacity in GPHAC. The importance of transcultural competence in GPHAC is becoming a consensus of public health professionals. China has extensively involved in enhancing global health cooperation with the world and other developing countries especially. This study, the first of its kind in China’s public health system demonstrated that, the training on transcultural capacity equipped the public health professional with adequate knowledge and awareness on transcultural competence and the need to adopt such attitude in global health practices as most collaborators and clients/patients are of diverse cultural background. This would invariably promote global health cooperation among countries.

## Data Availability

The datasets used during the current study are available from the first author or corresponding author on reasonable request.

## Abbreviations

China CDC: Chinese Center for Disease Control and Prevention
CDCs: Centers for Disease Control and Prevention
GPHAC: Global public health assistance cooperation
